# Dietary glycaemic index, glycaemic load and breast cancer risk: a systematic review and meta-analysis

**DOI:** 10.1038/sj.bjc.6604618

**Published:** 2008-08-26

**Authors:** H G Mulholland, L J Murray, C R Cardwell, M M Cantwell

**Affiliations:** 1 Cancer Epidemiology and Prevention Research Group, Centre for Clinical and Population Sciences, Queens University Belfast, Mulhouse Building, Royal Victoria Hospital Site, Grosvenor Road, Belfast, BT12 6BJ, UK

**Keywords:** glycaemic index, glycaemic load, breast cancer, meta-analysis

## Abstract

This systematic review aimed to examine if an association exists between dietary glycaemic index (GI) and glycaemic load (GL) intake and breast cancer risk. A systematic search was conducted in Medline and Embase and identified 14 relevant studies up to May 2008. Adjusted relative risk estimates comparing breast cancer risk for the highest versus the lowest category of GI/GL intake were extracted from relevant studies and combined in meta-analyses using a random-effects model. Combined estimates from six cohort studies show non-significant increased breast cancer risks for premenopausal women (relative risk (RR) 1.14, 95% CI 0.95–1.38) and postmenopausal women (RR 1.11, 95% CI 0.99–1.25) consuming the highest versus the lowest category of GI intake. Evidence of heterogeneity hindered analyses of GL and premenopausal risk, although most studies did not observe any significant association. Pooled cohort study results indicated no association between postmenopausal risk and GL intake (RR 1.03, 95% CI 0.94–1.12). Our findings do not provide strong support of an association between dietary GI and GL and breast cancer risk.

Glycaemic index (GI) values classify foods according to the 2-h blood glucose response after consuming a portion of the food containing 50 g of available carbohydrate, compared with the equivalent amount from a standard food, such as glucose or white bread (Jenkins *et al*, 1981). The glycaemic load (GL) concept was later developed to better reflect the blood glucose response and insulin demand of a food by taking into account the total amount of carbohydrate usually consumed in addition to its GI value (Salmeron *et al*, 1997).

Habitual consumption of a high GI or GL diet may promote carcinogenesis by inducing hyperglycaemia and hyperinsulinaemia (Brand-Miller, 2003), potentially acting through the insulin-like growth factor (IGF) axis (Biddinger and Ludwig, 2005). A recent meta-analysis illustrated that IGF-1 levels were associated with premenopausal but not postmenopausal breast cancer risk (Renehan *et al*, 2006). Additionally, high GI diets may promote weight gain (Brand-Miller *et al*, 2002). High body fatness contributes to an increased risk of postmenopausal, yet a reduced risk of premenopausal, breast cancer (Renehan *et al*, 2008). There has been a recent surge in research of the effect of GI and GL intake on breast cancer risk; however, results to date have been conflicting (Augustin *et al*, 2001; Key and Spencer, 2007; McCann *et al*, 2007). This may not be surprising, given the disparities between the biologically plausible mechanisms suggested above.

In this systematic review, we had the aim of clarifying any association between dietary GI, GL and breast cancer risk, and of determining if risk varies according to menopausal status or body fatness.

## Materials and methods

Ovid Medline, including Medline In-Process (US National Library of Medicine, Bethesda, MD, USA), and Embase (Reed Elsevier PLC, Amsterdam, Netherlands) databases were systematically searched for relevant studies published up to May 2008. The search strategy incorporated various medical search heading terms and keywords for GI, GL, nutrition and cancer. Animal studies were excluded but no language restrictions were imposed. The inclusion criteria included both cohort and case–control studies that had assessed dietary GI and/or GL intake in their study population and reported adequate information regarding cancer incidence, including relative risk (RR) estimates and corresponding 95% confidence intervals (CIs).

Two independent reviewers (HGM, MMC) screened studies for inclusion by examining abstracts and then full text where necessary, with discrepancies resolved by discussion. The reference lists of all included studies were also searched. The reviewers extracted information on study design, population characteristics, exclusion criteria, dietary assessment of GI and GL, adjustments for confounders and results from each study. The Newcastle-Ottawa quality assessment scale (www.lri.ca) was applied to all studies to consider factors such as selection of participants, comparability of studies, follow-up and ascertainment of exposure and outcome.

Meta-analyses were conducted using studies that compared categories of GI or GL intake to produce risk estimates and presented results separately by menopausal status. Studies categorised intake by quartiles (McCann *et al*, 2007; Sieri *et al*, 2007; Lajous *et al*, 2008) or quintiles (Augustin *et al*, 2001; Cho *et al*, 2003; Jonas *et al*, 2003; Frazier *et al*, 2004; Higginbotham *et al*, 2004; Holmes *et al*, 2004; Lajous *et al*, 2005; Silvera *et al*, 2005). Two studies could not be included in meta-analyses, as GI/GL intakes were only examined as continuous variables (Nielsen *et al*, 2005; Giles *et al*, 2006), and another study was excluded, as it did not present results separately by menopausal status (Levi *et al*, 2002). Adjusted RR estimates and 95% CI comparing the highest versus the lowest category of GI and GL intake were combined and weighted using a random-effects model. Sensitivity analysis was performed for cohort and case–control studies separately, premenopausal versus postmenopausal women, by body mass index (BMI) categories where possible, by dietary assessment methods used, by GI/GL values, by follow-up time for cohort studies, by quality scale score, by geographic variations and by systematically removing each individual study. Heterogeneity in each meta-analysis was investigated using the *χ*^2^ test and *I*^2^ statistic. Funnel plots of study relative risks plotted against their corresponding standard errors were assessed for asymmetry to test for publication bias. Statistical analysis was conducted using Intercooled STATA version 9.2 (StataCorp 2005, College Station, TX, USA).

## Results

The electronic database searches identified 464 publications, and 2 independent reviewers screened abstracts, titles then full text to select 51 articles, which were relevant for data extraction on GI, GL and risk of all cancers. Four articles were multiple publications from the same study and one further article did not provide sufficient information on their results, and hence were excluded. Of the remaining studies, 14 specifically referred to breast cancer risk (Augustin *et al*, 2001; Levi *et al*, 2002; Cho *et al*, 2003; Jonas *et al*, 2003; Frazier *et al*, 2004; Higginbotham *et al*, 2004; Holmes *et al*, 2004; Lajous *et al*, 2005, 2008; Nielsen *et al*, 2005; Silvera *et al*, 2005; Giles *et al*, 2006; McCann *et al*, 2007; Sieri *et al*, 2007), the characteristics of which are summarised in Table 1. Cohort studies accounted for 10, one of which was a retrospective design, and the remaining 4 publications were case–control studies. Eight studies originated from North and Central America, five from Europe and one from Australia.

Cohort studies scored more highly on the quality scale compared with population-based case–control studies, which in turn ranked higher than hospital-based case–control studies (Table 1). In case–control studies, cases were identified by histological confirmation, whereas cohort studies identified cases through linkage to cancer registries, self-report, medical record review or a combination of these methods. Food Frequency Questionnaires (FFQs) were used in all studies to assess habitual dietary intake, and two cohorts repeated dietary assessment at multiple time points and subsequently were able to calculate cumulative average GI/GL intakes (Cho *et al*, 2003; Holmes *et al*, 2004). Most studies sourced GI and GL values from International Tables (Foster-Powell and Miller, 1995; Foster-Powell *et al*, 2002), with the exception of Sieri *et al* (2007), who primarily used GI and GL values calculated from their local Italian foods. The majority of studies included age, BMI and energy intake in their adjusted analysis and all that were included in meta-analyses adjusted for women's reproductive and menstrual histories (Table 1). In addition, only two studies controlled for history of diabetes among breast cancer cases (Augustin *et al*, 2001; Jonas *et al*, 2003).

**Figure 1:**
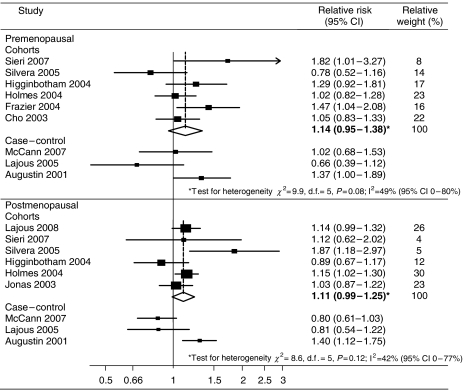
Forest plot of highest versus lowest category of GI intake and breast cancer risk. Bold relative risks denote combined effect estimates.

There was evidence of marked heterogeneity in analyses of GI/GL intake and breast cancer risk when all studies were combined and therefore analyses were restricted to cohort studies only. As shown in Figure 1, there was some evidence of an association between the highest versus the lowest category of GI intake and premenopausal (RR 1.14, 95% CI 0.95–1.38) and postmenopausal (RR 1.11, 95% CI 0.99–1.25) risk when six cohort study results were combined; however, these did not reach statistical significance. Although not statistically significant, moderate heterogeneity was still observed, which was not reduced when studies were grouped by differences in their quality scale score, cohort follow-up time, geographic variations or median GI/GL values. When analysis was restricted to cohort studies that had incorporated a more robust measure of dietary intake, that is, ⩾100-item FFQ, heterogeneity was somewhat reduced and a significant association emerged between GI intake and premenopausal (RR 1.20, 95% CI 1.01–1.43, *I*^2^=37%, *P*=0.17) and postmenopausal risk (RR 1.10 95% CI 1.02–1.19, *I*^2^=0%, *P*=0.46). Significant heterogeneity was observed when the results of case–control studies examining premenopausal (*I*^2^=64%, *P*=0.05) or postmenopausal risk (*I*^2^=83%, *P*<0.01) were combined and so the pooled estimate is not presented. However, only the hospital-based case–control study demonstrated a positive association between GI intake and breast cancer risk. None of the studies that were excluded from our meta-analyses demonstrated an association with GI intake, two of which were conducted in postmenopausal women and one that combined premenopausal and postmenopausal women (Levi *et al*, 2002; Nielsen *et al*, 2005; Giles *et al*, 2006).

**Figure 2:**
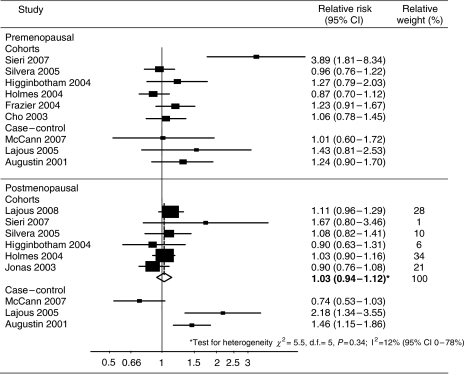
Forest plot of highest versus lowest category of GL intake and breast cancer risk. Bold relative risks denote combined effect estimates.

There was a lack of symmetry in the funnel plot of GL and premenopausal breast cancer, the results indicating possible publication bias. As shown in Figure 2, most studies did not demonstrate any evidence of an association between the highest versus the lowest category of GL intake and premenopausal risk, with one notable exception (Sieri *et al*, 2007). There was evidence of heterogeneity when combining cohort studies (*I*^2^=69%, *P*<0.01), and therefore no combined risk estimate is presented, but removing the study by Sieri *et al* revealed no association between GL and premenopausal risk (RR 1.02, 95% CI 0.89–1.16, *I*^2^=9%, *P*=0.35). Combining data from cohort studies demonstrated no evidence of an association between postmenopausal risk in the highest GL consumers (RR 1.03, 95% CI 0.94–1.12). Significant heterogeneity was incorporated when combining case–control studies, and therefore no combined estimate is presented, although results from these were inconsistent. Two studies examined GL as continuous variables but did not identify any significant association with breast cancer risk (Nielsen *et al*, 2005; Giles *et al*, 2006).

Five studies presented GI, GL and breast cancer risk results stratified by BMI categories (Cho *et al*, 2003; Holmes *et al*, 2004; Silvera *et al*, 2005; McCann *et al*, 2007) and the observed associations did not differ when these were combined separately for normal weight or overweight women (data not shown).

## Discussion

This systematic review and meta-analyses of GI and GL intake and breast cancer risk is the most comprehensive to date and the first to examine the association by menopausal status. Overall, we did not find any strong association between these dietary carbohydrate measures in relation to either premenopausal or postmenopausal risk.

Although no significant association was observed between the highest versus the lowest category of GI intake and breast cancer risk, positive associations became apparent once analysis was restricted to cohort studies utilising a more robust measure of dietary intake, that is, ⩾100-item FFQs. However, in our systematic review, we performed many stratified analyses to reduce statistical heterogeneity, and therefore any associations shown could have been due to chance. Furthermore, most studies that were not included in our meta-analysis did not observe significant associations with GI (Nielsen *et al*, 2005; Giles *et al*, 2006).

There was some evidence of publication bias when examining funnel plots of GL and premenopausal breast cancer risk, although the majority of studies reported no significant associations. Our findings also provided little evidence of an association between GL intake and postmenopausal risk. A recent meta-analysis of 20 studies demonstrated a 1.2-fold increase in risk for women with diabetes mellitus (Larsson *et al*, 2007), suggesting that hyperinsulinaemia may be a contributory factor in breast cancer. However, if risk is related to the overall insulin demand of the diet, stronger associations would be expected for GL rather than GI (Key, 2001) and we observed no association between GL and breast cancer risk. Additionally, C-peptide, a marker of insulin secretion, was not found to be related to risk in two well-designed cohort studies (Verheus *et al*, 2006; Eliassen *et al*, 2007), and so there is little evidence to support a direct association between insulin and breast carcinogenesis.

It has been hypothesised that chronic hyperinsulinaemia induced by a high GI diet may suppress fat oxidation and promote carbohydrate oxidation in the body, resulting in an enhanced appetite and body fat gain (Brand-Miller *et al*, 2002). We did not observe any association between GI/GL intake and breast cancer risk by BMI categories in our limited analysis. However, only five studies reported risk by BMI and none of these were powered to include obese women as a separate subgroup (Cho *et al*, 2003; Holmes *et al*, 2004; Silvera *et al*, 2005; McCann *et al*, 2007), therefore further research is warranted in this population subgroup.

It is possible that dietary measurement error associated with FFQs may have attenuated any real association between GI, GL and breast cancer risk. The FFQs used were quite variable in length, ranging from 61 items to 192 items in length and only two studies incorporated repeat dietary assessments at different time points to account for potential changes in dietary habits during the follow-up period (Cho *et al*, 2003; Holmes *et al*, 2004). Importantly, individual studies reported in this review collected information about menopausal status only at baseline, which after long follow-up periods, for example, in the Canadian Breast Screening Study and the US Nurses Health Study (Holmes *et al*, 2004; Silvera *et al*, 2005), are likely to be inaccurate at the time of analysis. Future studies should ideally collect information on BMI and menopausal status at multiple time points during follow-up periods.

Our meta-analysis has limitations; for example, the study results were inconsistently adjusted for potential confounders (e.g., history of diabetes), which might result in residual confounding. Each study in the meta-analyses had categorised GI and GL intake differently, and utilised a mixture of glucose and white bread reference values. Therefore, we assessed risk in the highest compared with the lowest category of intake, although absolute GI and GL intake within these categories differed between studies, which is not ideal. A relatively small number of studies were included in our analyses, particularly for subgroup analysis, which made it difficult to estimate publication bias and heterogeneity. Nevertheless this meta-analysis achieved reasonable statistical power. In conclusion, our systematic review suggests that high dietary GI and GL intakes do not appear to be of aetiological importance in breast tumour development.

## Figures and Tables

**Table 1:** Characteristics of studies included in systematic review of dietary glycaemic index, glycaemic load and risk of breast cancer

									**Adjusted confounders**											
**Authors (date), location**	**Study**	**Study design (mean follow-up)**	**Cases**	**Controls/cohort size**	**Diet assessment**	**Quality scale score**	**Median GI (IQ range)**	**Median GL (IQ range)**	**Age**	**BMI**	**Energy**	**Hormon.**	**Reprod.**	**Menstr.**	**Smoking**	**PA**	**Education**	**Alcohol**	**Family**	**BBD**
Lajous *et al* (2008), France	E3N Study	Prospective cohort (9 years)^a^	1812	62 739	Self-reported 208-item FFQ	9/9	55 (44–66)	123 (84–165)	✓	✓	✓	✓	✓	✓		✓	✓	✓	✓	✓
Sieri *et al* (2007), Italy	ORDET Study	Prospective cohort (11.5 years)	289	8959	Self-reported 107-item FFQ	8/9	56 (52–59)	113 (97–151)	✓	✓	✓	✓	✓	✓	✓		✓	✓		
McCann *et al* (2007), USA	WEB Study	Population-based case–control	1166	2105	Interviewed FFQ	7/9	77 (70–83)^b^	147 (104–186)^b^	✓	✓	✓		✓	✓			✓		✓	✓
Giles *et al* (2006), Australia	Melbourne Collaborative Cohort Study	Prospective cohort (9.1 years)	324	12 273	Self-reported 121-item FFQ	9/9	49 (46–53)	108 (77–150)	✓	#	✓	✓	#	#		#	#	#	#	
Nielsen *et al* (2005), Denmark	Diet, Cancer & Health Cohort	Prospective cohort (6.6 years)	634	23 870	Self-reported 192-item FFQ	9/9	—	—	✓	✓		✓	✓				✓	✓		
Silvera *et al* (2005), Canada	National Breast Screening Study	Prospective cohort (16.6 years)	2518	49 111	Self-reported 86-item FFQ	9/9	77 (60–96)	104 (83–123)	✓	✓	✓	✓	✓	✓				✓	✓	✓
Lajous *et al* (2005), Mexico		Population-based case–control	475	1391	Interviewed FFQ	7/9	62 (—)	152 (44–214)	✓	✓	✓		✓	✓					✓	
Higginbotham *et al* (2004), USA	Women's Health Study	Prospective cohort (6.8 years)	946	38 446	Self-reported 131-item FFQ	9/9	53 (50–55)	117 (92–143)	✓	✓	✓	✓	✓	✓	✓	✓		✓	✓	
Holmes *et al* (2004), USA	Nurses’ Health Study	Prospective cohort (18 years)	4092	88 678	Multiple self-reported 61+ item FFQs	8/9	75 (69–81)	105 (81–130)	✓	✓	✓		✓	✓				✓	✓	✓
Frazier *et al* (2004), USA	Nurses’ Health Study II	Retrospective cohort	361	47 355	Self-reported 131-item FFQ	8/9	79 (74–84)	170 (141–202)	✓	✓	✓	✓	✓	✓				✓	✓	✓
Cho *et al* (2003), USA	Nurses’ Health Study II	Prospective cohort (8 years)^a^	714	90 655	Self-reported 133-item FFQ 142-item FFQ	8/9	77 (70–82)	120 (97–148)	✓	✓	✓	✓	✓	✓	✓			✓	✓	✓
Jonas *et al* (2003), USA	CPS II Nutrition Cohort	Prospective cohort (5 years)	1442	63 307	Self-reported 68-item FFQ	8/9	74 (65–85)	81 (58–103)	✓	✓	✓	✓	✓	✓	✓	✓	✓	✓	✓	✓
Levi *et al* (2002), Switzerland		Hospital-based case–control	331	534	Interviewed 79-item FFQ	6/9	92 (73–112)	—	✓		✓	✓	✓	✓		✓	✓	✓		
Augustin *et al* (2001), Italy		Hospital-based case–control	2569	2588	Interviewed 78-item FFQ	6/9	74 (70–79)	132 (98–174)	✓		✓	✓	✓	✓		✓	✓	✓		

CPS=Cancer Prevention Study; E3N=French component of European Prospective Investigation into Diet and Cancer Study; ORDET=Hormones and Diet in Etiology of Breast Tumors Study; WEB=Western New York Exposure and Breast Cancer Study.

a Total follow-up length, mean not reported.

b Postmenopausal GI/GL data; majority of study participants (60–70%) are postmenopausal.

Adjusted confounders: age; BMI=body mass index or body weight; energy=energy intake; hormon.=hormone replacement therapy/oral contraceptive use; reprod.=reproductive factors (e.g., parity, age at first birth); menstr.=menstrual history (e.g., age at menarche or menopause, menopausal status); smoking; PA=physical activity; education; alcohol=alcohol intake; family=family history of breast cancer; BBD=history of benign breast disease. ^#^ confounder tested but not included in final model.
